# Colchitaxel, a coupled compound made from microtubule inhibitors colchicine and paclitaxel

**DOI:** 10.1186/1860-5397-2-13

**Published:** 2006-06-30

**Authors:** Karunananda Bombuwala, Thomas Kinstle, Vladimir Popik, Sonal O Uppal, James B Olesen, Jose Viña, Carol A Heckman

**Affiliations:** 1 Department of Chemistry, Bowling Green State University, Bowling Green, OH 43403, USA; 2 Center for Microscopy & Microanalysis, Bowling Green State University, Bowling Green, OH 43403, USA; 3 IFF Research & Development, Union Beach NJ 07735, USA; 4 Department of Chemistry, University of Georgia, Athens, GA 30602-2556, USA; 5 Biology Department, Ball State University, Muncie, IN 47306, USA; 6 Scientific Volume Imaging BV, Alexanderlaan 14, 1213 XS Hilversum, The Netherlands

## Abstract

**Background:**

Tumor promoters enhance tumor yield in experimental animals without directly affecting the DNA of the cell. Promoters may play a role in the development of cancer, as humans are exposed to them in the environment. In work based on computer-assisted microscopy and sophisticated classification methods, we showed that cells could be classified by reference to a database of known normal and cancerous cell phenotypes. Promoters caused loss of properties specific to normal cells and gain of properties of cancer cells. Other compounds, including colchicine, had a similar effect. Colchicine given together with paclitaxel, however, caused cells to adopt properties of normal cells. This provided a rationale for tests of microtubule inhibitor combinations in cancer patients. The combination of a depolymerizing and a stabilizing agent is a superior anti-tumor treatment. The biological basis of the effect is not understood.

**Results:**

A single compound containing both colchicine and paclitaxel structures was synthesized. Colchicine is an alkaloid with a trimethoxyphenyl ring (ring A), a ring with an acetamide linkage (ring B), and a tropolone ring (ring C). Although rings A and C are important for tubulin-binding activity, the acetamide linkage on ring B could be replaced by an amide containing a glutamate linker. Alteration of the C-7 site on paclitaxel similarly had little or no inhibitory effect on its biological activity. The linker was attached to this position. The coupled compound, colchitaxel **(1)**, had some of the same effects on microtubules as the combination of starting compounds. It also caused shortening and fragmentation of the + end protein cap.

**Conclusion:**

Since microtubule inhibitor combinations give results unlike those obtained with either inhibitor alone, it is important to determine how such combinations affect cell shape and growth. Colchitaxel shows a subset of the effects of the inhibitor combination. Thus, it may be able to bind the relevant cellular target of the combination. It will be useful to determine the basis of the shape reversal effect and possibly, the reasons for therapeutic efficacy of microtubule inhibitor combinations.

## Background

Colchicine **(2)** is a major alkaloid found in the plant, *Colchicum autumnale*. It has antiinflammatory and anticancer properties and is used in the treatment of gout [1]. Colchicine binds tightly to the tubulin subunits that compose the microtubule. Tubulin is a dimeric protein of 110,000 Daltons consisting of two non-identical chains of 55,000 Daltons (α and β). Tubulin subunits polymerize in a linear fashion to make up a protofilament, and 13 protofilaments in a circular arrangement make up the microtubule. The microtubule can undergo assembly and disassembly at both ends. The structure has a directionality, however, which is dictated by the kinetics of subunit addition and subtraction. The end that undergoes rapid assembly is the plus (+) end. Colchicine and a number of other compounds, including podophyllotoxin and vinca alkaloids, e.g. vinblastine, serve as a + end cap. Under physiological conditions, colchicine prevents subunit addition at the + end, and the structure eventually depolymerizes from the minus (-) end [2–3].

Paclitaxel **(7)** is a compound originally isolated from the bark of the pacific yew tree (*Taxus brevifolia*). Its registered name is Taxol. When thousands of plant extracts were screened for biological activity by the National Cancer Institute and U. S. Department of Agriculture, one of the extracts, from the pacific yew, blocked cell division. Following isolation and purification, the structure was determined [4]. Paclitaxel decreased the magnitude of the dissociation constant for tubulin at both the + and - ends of the microtubule [5], thereby promoting microtubule assembly and reducing the amount of free tubulin in the cell [6–7]. The amino-terminal domain of tubulin, extending up to residue 205, forms a Rossmann fold in which parallel β-strands alternate with four α-helices. The next domain contains five helices and a mixed β-sheet, one strand of which is contiguous to the β-sheet in the amino-terminal domain. Nogales and coworkers showed that the α-subunit resembles the β- and is stacked on it in such a way that the nucleotide-binding site is embedded in the interface [8]. Ravelli and coworkers found colchicine binding site to be buried in the β subunit, boxed in by β-strands of the second domain and helices #7 and #8. The A ring of colchicine contacts residue 241[9], consistent with previous evidence of binding at Cys(239) [10]. The former workers also suggested that the reason for the destabilizing effect of colchicine on polymerized tubulin was that colchicine binding displaced the M loop. This loop is situated between the binding faces of two protofilaments and is therefore important for lateral binding between tubulin dimers [11].

The anti-tumor activity of microtubule inhibitors is often attributed to their ability to inhibit cell division. When the cell starts to divide, microtubules are organized into two arrays, each having the - ends associated with one of two microtubule organizing centers located near the cell periphery and the + ends located towards the chromosomes. This enables the microtubules to pull the daughter chromosomes apart during mitotic division. However, microtubules also play important roles in organelle transport, cell shape maintenance, and motility. The discovery of the potent anti-tumor effect of paclitaxel and its semi-synthetic analogue, docetaxel, stimulated a search for novel substances that interact with tubulin. Since the vinca structure allowed only minor alterations and the potencies of colchicine derivatives were rather low, recent interest has been focused on new, highly-active classes of natural products such as the dolastatins and the cryptophycins. Such natural compounds and their synthetic derivatives have been evaluated for dosage and side effects in preliminary clinical trials [12–15], but the results do not suggest that they have potent anticancer activity. Although the inhibitor combinations are thought to be effective because they interfere with chromosome separation by two separate microtubule-related mechanisms, there is also evidence that another mechanism of synergy may exist (see Discussion).

In an assay for three-dimensional cell phenotypes, based on computer-assisted microscopy and sophisticated classification methods, shape data from treated cells were classified by reference to a cell line that became oncogenically transformed over a prolonged course of in vitro culture. Colchicine-treated cells shifted their shape phenotype to one resembling that of cancer cells [16]. When supplied along with paclitaxel, however, colchicine shifted the phenotype to one resembling normal cells [17]. Similar combinations of microtubule inhibitors, made up of docetaxel with microtubule depolymerizing agent, 5'-noranhydrovinblastine, also called vinorelbine, are effective in anticancer therapy [18–27]. The therapy is only effective if the timing of administration of the agents was as close as possible, and objective response was greater than if either microtubule inhibitor was used alone (reviewed in [28–29]). The cooperative interaction of the microtubule inhibitors is not well understood, as mentioned above, but is difficult to study because microtubule length, flexibility, bundling, and anchorage in the microtubule organizing center are affected in different ways by the single inhibitors and their combination. Methods of quantifying these endpoints are lacking, so that, with few exceptions comparisons must be based upon qualitative data. The laboratory's classification procedure addresses this deficiency, as it allows phenotypes created in an experiment to be related to a database of known normal and oncogenically transformed cells. Moreover, specific features of the cancer- and normal-type cells, such as filopodia, arm-like protrusions, rounding-up, and numerous aspects of vesicle trafficking, can be recognized [30].

Colchicine is a tricyclic alkaloid. The third ring, ring C, is essential for microtubule binding activity, as change of the keto group to a thioketo group or demethylation of the OMe group in this ring reduced the activity [31](reviewed in [2]). Demethylation of the OMe group in ring A also decreased the activity, indicating the importance of this ring for binding. However, the acetamide linkage on ring B could be replaced by other alkyl amides with little change in potency [32]. Moreover, colchicine with an altered B ring still bound tubulin [33]. The binding site of paclitaxel is situated on the inner face of the polymerized microtubule, tucked into a bend formed by helix #6 and helix #7 near the + end of the microtubule and adjacent to strand #7. Here, the taxane may affect both the structure of the M loop and that of helix #6 which contacts the GTP binding site, so that it may both stabilize GTP against hydrolysis and stabilize the M loop against curvature on one side. Since the interaction between domains is very tight, an anti-microtubule drug can affect the dynamic assembly and instability of the whole microtubule. The effect of simultaneous colchicine and paclitaxel treatment is complicated by the fact that they bind to adjacent sites on tubulin. Indeed, this may contribute to the difficulty of understanding the effects of the drug combination (see below).

In the synthesis of colchitaxel, the acetamide on ring B was replaced by an amide containing a glutamate linker. If a site could be identified on paclitaxel, which had little or no effect on the biological activity of the compound, the linker could be attached there. C-7 was apparently on the part of the molecule not engaged in tubulin binding, since modification was attempted previously and shown not to interfere with paclitaxel's ability to inhibit tumor cell growth [34]. The linker was attached at this site. The current experiments show that the novel compound, colchitaxel, retains the ability to bind microtubules and affects the + end cap structure when administered to intact cells. The coupled compound will help investigators determine the reasons for the therapeutic efficacy of microtubule inhibitor combinations.

## Results and Discussion

### Synthetic Approach

**Scheme 1:**
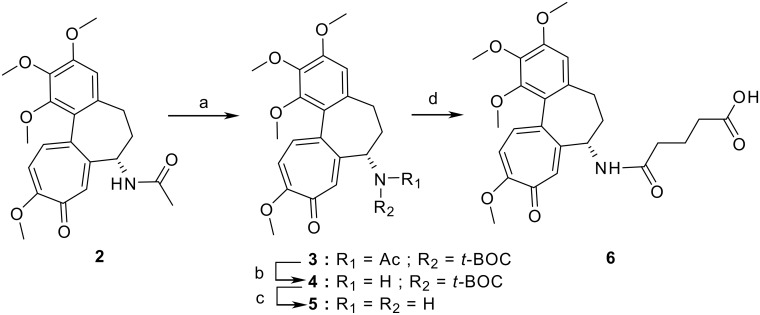
Reagents and conditions for synthesis of *N*-glutaryl-deacetylcolchicine. The reagents used at each step are: a) DCC, DMAP, Et_3_N; b) MeONa at 0°C; c) TFA; d) glutaric anhydride, dimethylformamide (DMF)

**Scheme 2:**
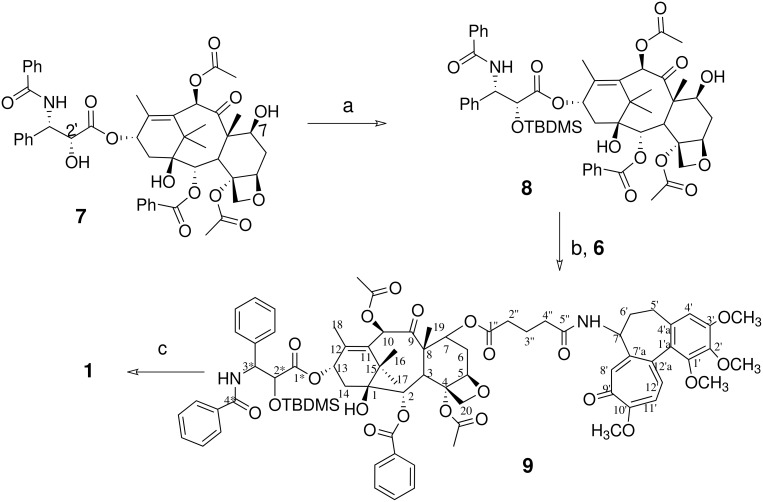
Reagents and conditions for protection of paclitaxel and coupling to *N*-glutaryl-deacetylcolchicine **(6)**. The reagents used at each step are: a) TBDMSCl, DMAP; b) DCC, DMAP; c) TBAF.

Colchitaxel **(1)** was made by coupling two agents, paclitaxel and colchicine, with a glutarate linker. Hydrolysis of the acetamide group in colchicine **(2)** provides *N-*deacetylcolchicine **(5)**, which contains a reactive amino group (Scheme 1). The latter can be derivatized with little change in the biological properties of the drug [35]. In paclitaxel **(7)** there are two secondary hydroxy groups which may be used for the attachment of a linker. The steps in attachment are described in Scheme 2. The 2' group, however, is essential for paclitaxel binding to tubulin. This group needed to be protected during derivatization.

Since acid hydrolysis of the acetamide group in **(2)** also results in a loss of the methyl group on the adjacent tropolone ring [36], the method of Lebeau and coworkers was used to transform the amide group into the corresponding *t*-butyl carbamate group [37]. This allowed the acetyl substituent to be removed easily and quantitatively [38]. The amide nitrogen in colchicine **(2)** was protected with a *tert*-butoxycarbonyl (*t-*Boc) group by treatment with an excess of di(*tert*-butyl)dicarbonate (DCC) in the presence of 4-dimethylaminopyridine (DMAP) and triethylamine (Et_3_N). *N*-(*t*-Boc)colchicine **(3)** obtained in this reaction was then deacetylated using a methanolic solution of sodium methoxide (MeONa) to give *N*-(*t*-Boc)deacetylcolchicine **(4)**. Treatment of **4** with trifluoroacetic acid (TFA) under mild conditions results in removal of the *t*-Boc group and formation of deacetylcolchicine **(5)**. Reaction of the latter with glutaric anhydride produces *N*-glutaryl-deacetylcolchicine **(6)**.

The 2'-OH group in paclitaxel **(7)** was protected as a *tert*-butyldimethylsilyl (TBDMS) ether using a slight modification of a previous method [34]. The coupling of 2'-O-TBDMS-paclitaxel **(8)** with *N*-glutaryl-deacetylcolchicine **(6)** was conducted employing the procedure described by Postema and co-workers [39]. The removal of TBDMS protection from 2'-OH group was achieved using tetra-*N*-butylammonium fluoride (TBAF) as described in a previous report [40]. The reactions are summarized in Scheme 2.

### Estimation of Partition Coefficients

In order to estimate the extent to which colchitaxel may be able to permeate the cell membrane, logP values computed for the new compound were compared with those of the starting compounds. Its logP values were somewhat higher but closely resembled those computed for paclitaxel (Table 1). This suggested that the compound would show a similar ability to permeate into cells as paclitaxel.

**Table 1:** Comparison of the logP values calculated by several methods

Program	Compound		

	colchicine	paclitaxel	colchitaxel

ALOGP	1.59	3.20	4.64
CLOGP	1.20	4.95	6.50
IA_logP	1.72	1.73	5.53
KowWin	1.86	3.31	--
LogD Suite	1.03 ± 0.5	7.24 ± 0.81	8.58 ± 0.88

Modeling software was used to estimate logP values for colchicine, paclitaxel, and the newly synthesized compound, Colchitaxel. The top calculations were performed in ALOGPS 2.1 software (Virtual Computational Chemistry Laboratory). Other programs used were from Syracuse Research Corporation (KowWin), IALogP (Interactive Analysis), Daylight Chemical Information Systems, Inc. (ClogP), and ACD (LogD Suite). The only compound which had a measured logP value was colchicine (value 1.30).

### Effects of Colchitaxel on Microtubules

**Figure 1:**
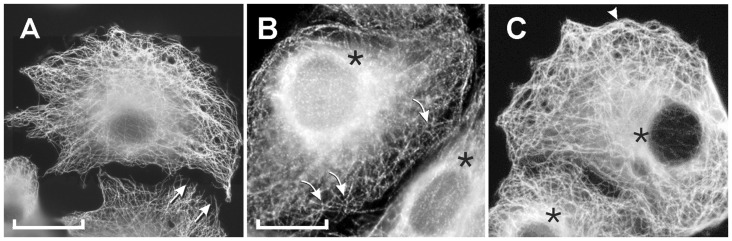
Microtubule arrangement as visualized by immunofluorescence localization of β-tubulin. Cells were treated with: A) 2 μM Colchitaxel, B) 12 μM Colchitaxel, or C) solvent vehicle alone. With the low concentration of compound, microtubules occasionally appear to be arranged perpendicular to the cell edge (arrows). With higher concentrations, cytoplasmic foci are observed with radiating microtubules (curved arrows). In addition, the region around the centrosome (asterisks) is more evenly stained in treated cells. Control cells typically show a concentration of staining at one side of the nucleus. In control cells, the microtubules usually bend to run parallel to the edge (arrowhead). A) bar = 25 μm, B) and C) bar = 10 μm.

Further studies were performed to evaluate the biological effects of the novel compound. Cells were exposed to varying concentrations of colchitaxel, and then the microtubules were localized in order to determine whether their structure or distribution was affected. Microtubules were occasionally found to be oriented perpendicular to the cell edge (Figure 1), a pattern previously observed in cells exposed simultaneously to colchicine and paclitaxel [17]. In control cells, the microtubules typically curved so that their distal ends ran parallel to the cell edge. In cells treated with colchitaxel at high concentrations, the microtubules were less robust, although their structural integrity was conserved up to a concentration of 12 μM. These cells also showed 'x' shaped cytoplasmic foci from which microtubules appeared to radiate. These arrangements were also observed in cells treated with the combination of microtubule inhibitors [17]. Bundles of microtubules, which had characterized cells treated with the combination of starting compounds, were not formed in colchitaxel-treated cells (Figure 1).

**Figure 2:**
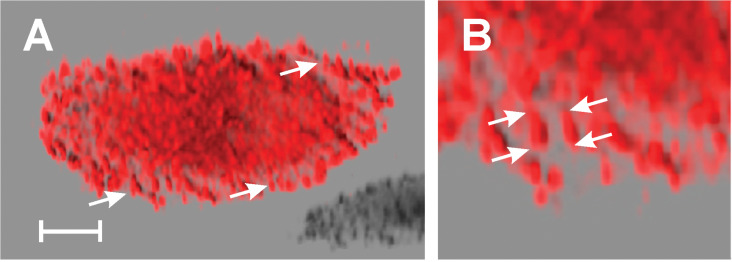
Projections of a VRO showing + ends localized by antibody against EB1 in a control cell. Cell was treated with solvent vehicle only. A) When viewed from the underside, the cell shows finger-like structures directed downward toward the substratum (arrows). B) When viewed at higher magnification, the EB1-positive ends appear elongated and smooth. The typical comets' lengths are designated by the arrows. A) bar = 5 μm, B) length between parallel arrow tips is between 6 and 7 μm.

**Figure 3:**
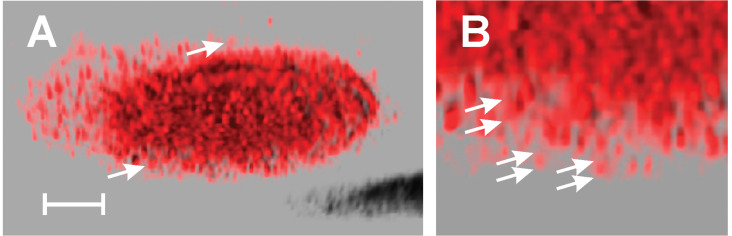
Projections of a VRO showing + ends localized by antibody against EB1 in colchitaxel-treated cell. The cell was treated with 6 μM Colchitaxel for 2 h. A) When viewed from the underside, the cell shows short dot-like structures which are particularly obvious at the edge (arrows). B) When viewed at higher magnification, the EB1-positive ends appear compact. The typical comets' lengths are designated by the arrows. A) bar = 5 μm, B) length between parallel arrow tips is between 3.5 and 4.5 μm.

An additional method of analysis was employed to determine whether EB1 proteins marking the + end of the microtubules were affected by colchitaxel. EB1 displays appeared as 'comets' in the virtual reality object (VRO) made from a three-dimensional image. The VROs reconstructed from laser confocal scanning microscopy are shown in Supplemental data. In both untreated and treated cells, the + end 'comets' were concentrated near the cell edge and descended in arc-like patterns toward the substratum. In untreated cells, the + ends appeared like arrangements of finger-like structures when viewed from the underside of the cell (Figure 2). In treated cells, the 'comets' appeared more compact (Figure 3).

### Combined Paclitaxel and Colchicine Effects on Microtubules

**Figure 4:**
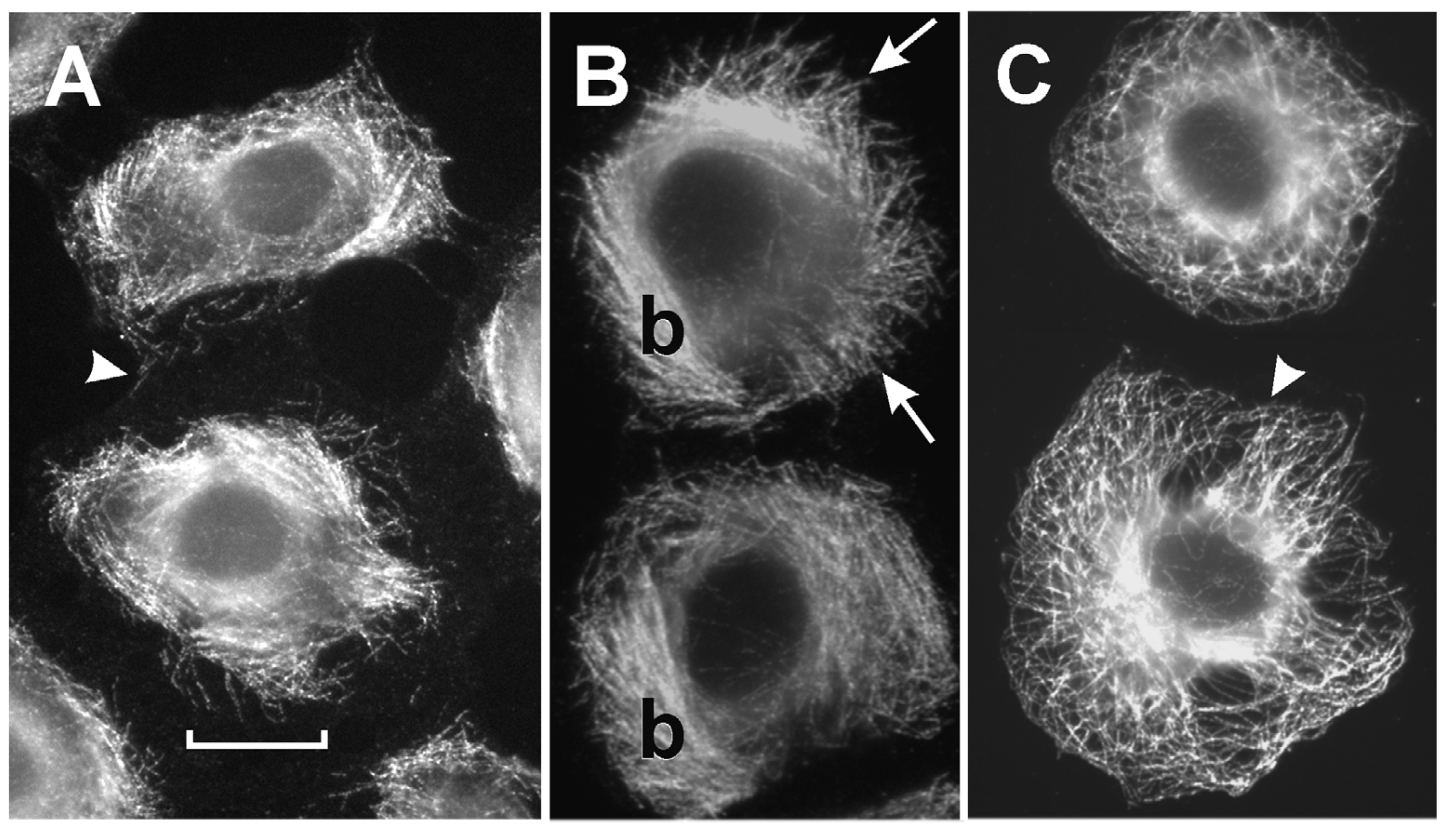
Microtubule arrangement as visualized by immunofluorescence localization of β-tubulin. Cells were treated with: A) 2 μM each colchicine and paclitaxel, B) 6 μM paclitaxel and 2 μM colchicine, or C) solvent vehicle alone. With equimolar levels of the compounds, microtubules are slightly fragmented and retracted from the cell edge, which is indicated by the arrowhead. They are typically arranged perpendicular to the cell edge. In cells treated with a 3:1 ratio of paclitaxel:colchicine, the microtubules appear rigid (arrows) and form large bundles (b) in the cytoplasm. Their orientation also tends to be perpendicular to the edge. In control cells, the microtubules show a wavy pattern, typically bending and running parallel to the edge (arrowhead). A), B), and C) bar = 10 μm.

In order to expand the results previously described for single agents [17], cells treated with paclitaxel and colchicine in differing molar ratios were compared with those exposed to colchitaxel. If an equimolar concentration of agents was used, the periphery was free of microtubules indicating that they were depolymerized in this zone of cytoplasm (Figure 4A). As microtubules were retracted from the periphery, they appeared to concentrate in the center of the cell. The remaining microtubules appeared unusually rigid and frequently extended straight out toward the cell edge (Figure 4A). Cells treated with a 3:1 ratio of paclitaxel:colchicine appeared to have the majority of tubulin in microtubules and contained prominent bundles of microtubules. Both these cells and those treated with equimolar levels showed parallel microtubules arranged perpendicular to the cell edge (Figure 4B). As previously reported, cells treated with single agents individually showed complete polymerization and depolymerization after paclitaxel and colchicine exposure, respectively [17].

The conventional view of microtubule inhibitors as chemotherapeutic compounds is that they halt chromosome separation, and this causes the cells to enter a cell death pathway. At substoichiometric ratios to tubulin, both paclitaxel and microtubule-depolymerizing agents restrict periods of growth and shrinkage of the microtubule [43–44] (reviewed in [45]). Thus, synergy at the molecular level could be due to the two classes of agents affecting dynamicity through different mechanisms. Even when two microtubule-polymerizing agents were used, synergistic inhibition of microtubule dynamicity could be observed [46]. Paclitaxel and discodermolide also synergistically affected G2-M arrest, proliferation, and apoptosis [46]. Some workers speculate that such synergy might arise from the different binding affinities of the two molecules for different tubulin isotypes present in the microtubule. Another study of three microtubule polymerizing agents confirmed that paclitaxel and discodermolide had complementary effects, whereas eleutherobin and epothilone B could substitute for paclitaxel [47]. Considering the complexity of interrelationships among various signaling and cell death pathways, however, it is easy to imagine that changes in dynamicity affect other microtubule-mediated processes in addition to mitotic division. Indeed, Suyama and coworkers showed that inhibition of the extracellular regulated kinase (ERK) blocks the cytotoxic effect of paclitaxel and the accumulation of sub-G1 cells [48], suggesting that the ERK pathway is essential to the mechanism of cell killing.

Acceptance of the mechanism hypothesized above as a basis for the synergy has been limited, because results from other laboratories have suggested that cell survival is higher in cells treated simultaneously with vinblastine and paclitaxel [49]. Giannakakou and coworkers found that a schedule of alternating 2-day exposures to each agent separately assured synergy in cell killing, whereas the agents in combination had antagonistic effects. Other researchers found that the antiproliferative effect of paclitaxel was relatively little affected by the presence of microtubule depolymerizing agent, N-acetylcolchinol [50]. Workers who studied cells cultured in vitro found high IC50 levels, in the 10–50 nM range, for antiproliferative activity of paclitaxel and vinorelbine individually, but synergy with concentrations as low as 3 nM and 0.01 nM respectively [51]. Thus, differing model systems yielded results variously suggesting antagonistic, non-additive, or synergistic effects. However, there is evidence that cells enter apoptosis shortly after paclitaxel exposure, suggesting that they are directed into a cell death pathway before entering the mitotic phase of the cell cycle [52–53]. Conversely, certain cells treated at high concentrations could pass through mitosis but still avoid apoptosis. This was particularly true of rodent cell lines. These cells escape mitosis and reconstitute a G1 population with a tetraploid chromosome complement when the spindle cannot be formed, whereas human cells tend to remain in mitotic arrest indefinitely [54–55].

Although the cure rates obtained with combinations were superior to those obtained with single agents in rodent models, this was attributed to the fact that high levels of vinblastine could be delivered if paclitaxel was also present [56]. Clinical use of the combination does not generally rely on higher dosages than employed in treatment with the single drugs, however, suggesting that elevation of vinorelbine into ranges that were formerly toxic does not apply to results on humans. Thus, the data again suggest that the rodent model fails to conform to results obtained in actual practice.

Treatment with two-inhibitor combinations exerts complex effects on the microtubule array. It decreases the tendency of microtubules to be anchored in the microtubule organizing center [57] and causes rearrangement into patterns that are rarely found in untreated cells. Such rearrangements included parallel arrays at the cell edge and focal points in the cytoplasm from which microtubules radiate [17]. These arrangements have been found after treatment with colchitaxel, but the microtubules appeared less rigid in this case than in cells treated with the combination of starting compounds. Since reduction of the EB1 cap of the microtubule was observed in colchitaxel-treated cells, the compound appeared to compete for the EB1-binding site. Since EB1 is thought to activate addition of subunits on the + end of the microtubule, the latter effect might be related to the reduced dynamicity of the microtubule.

The above results suggest that the mechanism of cooperative action of the two starting compounds remains unexplained. Effects measured in this laboratory's assay were obtained over short periods of treatment with high concentrations of compound. The results obtained with the assay and classification procedure equate the extreme cancer cell phenotype with that induced transiently by promoter treatment [58]. The phenotype induced by the microtubule inhibitor combination is qualitatively similar to that of promoter-treated cells but differs in direction. Therefore, reversal of cancer cell phenotype suggested that the same downstream mechanisms, i.e. the activation or inhibition of protein kinase C-mediated networks, is implicated in both promotion and microtubule inhibitor combination effects. Like the apoptotic endpoint described above, effects in the shape assay were found after only a brief exposure to the agents. Although phenotype reversal could well rely upon inhibition of microtubule dynamicity, it could not be working at the level of microtubule integration with the kinetochore. Retardation of cell division could not be detected over so short a time as 2 h. Thus, an explanation of the synergy between microtubule inhibitors awaits the demonstration of a mechanism.

In past studies, it has been difficult to determine which, if any, aspects of microtubule dynamicity or reorganization were related to the therapeutic efficacy of inhibitor combinations. Although cell phenotype studies are suggestive of a synergy between microtubule inhibitors, it is more difficult to distinguish synergistic and additive effects in the clinical anticancer studies. The purpose of the present research was to make a single agent from paclitaxel and colchicine, which could be used to determine whether a single agent could have the same effect as the combination of agents. Indeed, the coupled agent retained some of the effects of the combination of starting agents but had fewer effects on microtubule structure. Future investigation of colchitaxel-treated cells by the shape assay will be useful in determining whether the microtubule rearrangements coincide with the reversal of the properties of cancer cells.

## Experimental

### General Analytical Procedures

Paclitaxel was obtained from Dabur, Inc., India. Colchicine was purchased from Acros Organics (Ceel, Belgium). Both were judged >97% pure after assessment by NMR, HPLC, and verification of m.p. All melting points were obtained using Mel-Temp apparatus. Proton and carbon NMR spectra were obtained in a 400 MHz Varian Unityplus NMR spectrometer. Tetramethylsilane (TMS) was used as an internal reference for NMR. IR spectra were recorded using a Thermo Nicolet IR 200 spectrophotometer. Mass spectra were recorded using a Shimadzu QP5050A/GC-17A instrument. All solvents were distilled prior to use. Flash column chromatography was carried out with Merck silica gel (60–200 Mesh size). Air-sensitive reactions were carried out under Ar or N_2_. The final product was stored under Ar at -70°C. Commercial programs, ClogP (BioByte, Claremont, CA) and LogD Suite from Advanced Chemistry Development (Toronto, Canada), were used to predict the partition coefficient of the final coupled compound **(1)**.

### Cell Culture

The IAR20 PC1 line, derived from the liver of inbred BD-VI rats, was available from ATCC (Manassas, VA). These cells were routinely grown in William's E medium supplemented with penicillin, streptomycin, 50 units/ml each, and 10% fetal bovine serum as described elsewhere [59]. Cells were subcultured weekly at a density sufficient to attain confluency within one week. The original standard curve relating cell shape features to cancer was developed by sampling and analyzing cells at various times over a time course of approximately 8 months [60–61].

### Indirect Immunofluorescence Localization

For immunofluorescence localization studies, cells were subcultured onto glass coverslips and left for 18–48 h to attach. They were treated with various compounds for 2 h. The coverslips were collected by immersion in methanol at -20°C. N357 monoclonal antibody against β-tubulin (Amersham Biosciences, Piscataway, NJ) was diluted 1:600 for use in microtubule localizations. Mouse antibody against EB1, obtained from BD Transduction Laboratories (San Francisco, CA), was diluted 1:100. As secondary antibodies, FITC-conjugated (U. S. Biochemical Corp., Cleveland, OH) or Alexa 488-conjugated goat anti-mouse immunoglobulin G (Molecular Probes, Eugene, OR) were used as described previously [17]. Wide-field fluorescence images were recorded on a Zeiss Axiophot microscope equipped with a 63× Neofluor lens. Images were acquired from a Roper Scientific RTE/CCD camera (Tucson, AZ) and PC running MetaMorph 4.6r5 software (Universal Imaging Corp., Buckinghamshire, UK).

### Laser Confocal Scanning Microscopy (LCSM) and Visualization

LCSM of the samples was performed on a Zeiss Pascal confocal microscope equipped with a 63× Neofluor lens. Software used for deconvolution included the Volocity (Improvision, Coventry, UK) and Huygens (Advanced Volume Imaging, Hilversum, The Netherlands) packages. VROs were made in VRWorx 2.5 software (VRToolbox, Pittsburgh, PA). The resulting three-dimensional images are displayed in Quickview on Apple Macintosh or Dell PC microcomputers.

## Supporting Information

File 1 Attached Proton Test: Spectrum of Attached Proton Test.

File 2 Mass Spectrum: Spectrum from Electron Spray Ionization Mass Spectrometry.

File 3 Proton NMR: Spectrum from Proton Nuclear Magnetic Resonance.

File 4 VRO showing microtubule + ends localized by antibody against EB1 in a control cell. Part of the VRO is rendered in a two-dimensional form in Figure 2. Control cells were treated with solvent vehicle only. Note that + ends of microtubules at the cell edge are elongated and point downward toward the substratum. The frame is 74 μm wide.

File 5 VRO showing microtubule + ends localized by antibody against EB1 in a colchitaxel-treated cell. Part of the VRO is rendered in the two-dimensional form in Figure 3. Note that + ends of microtubules at the cell edge appear blunt and rough, and EB1 distribution is occasionally discontinuous. The frame is 63 μm wide.

File 6 High-resolution MS of colchicine.

File 7 High-resolution MS of paclitaxel. (Top) Spectrum of starting material. (Bottom) Theoretical spectrum of paclitaxel sodium salt

File 8 Experimental data (DOC) containing all experimental data
