# Supplementary figures and images for: Colchitaxel, a coupled compound made from microtubule inhibitors colchicine and paclitaxel
Source: Beilstein J Org Chem. 2006 Jun 30;2:13. doi: 10.1186/1860-5397-2-13 (PMC1557522; doi:10.1186/1860-5397-2-13)

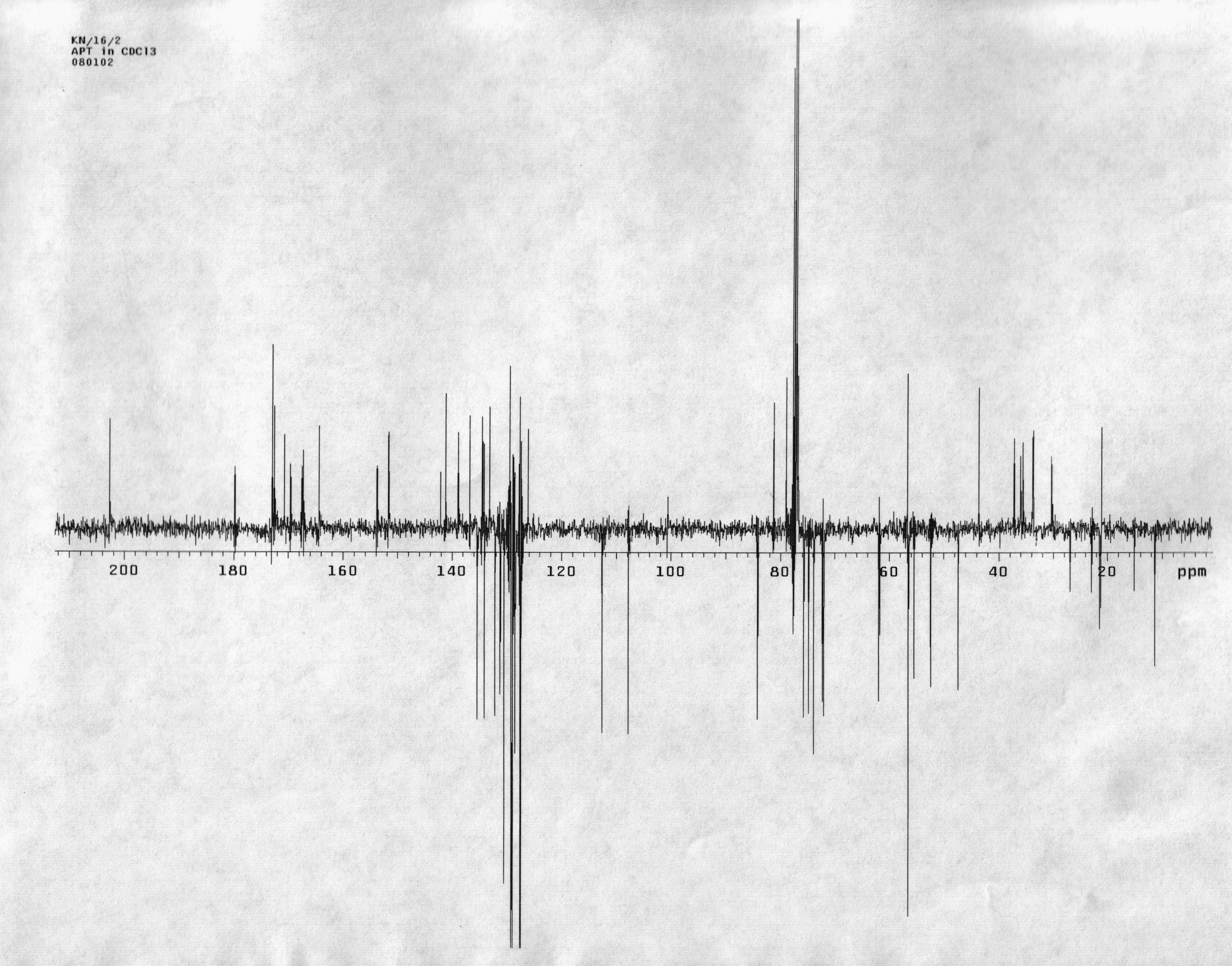

Supplement: File 1 — Attached Proton Test: Spectrum of Attached Proton Test. [file Beilstein_J_Org_Chem-02-13-s001.jpeg]

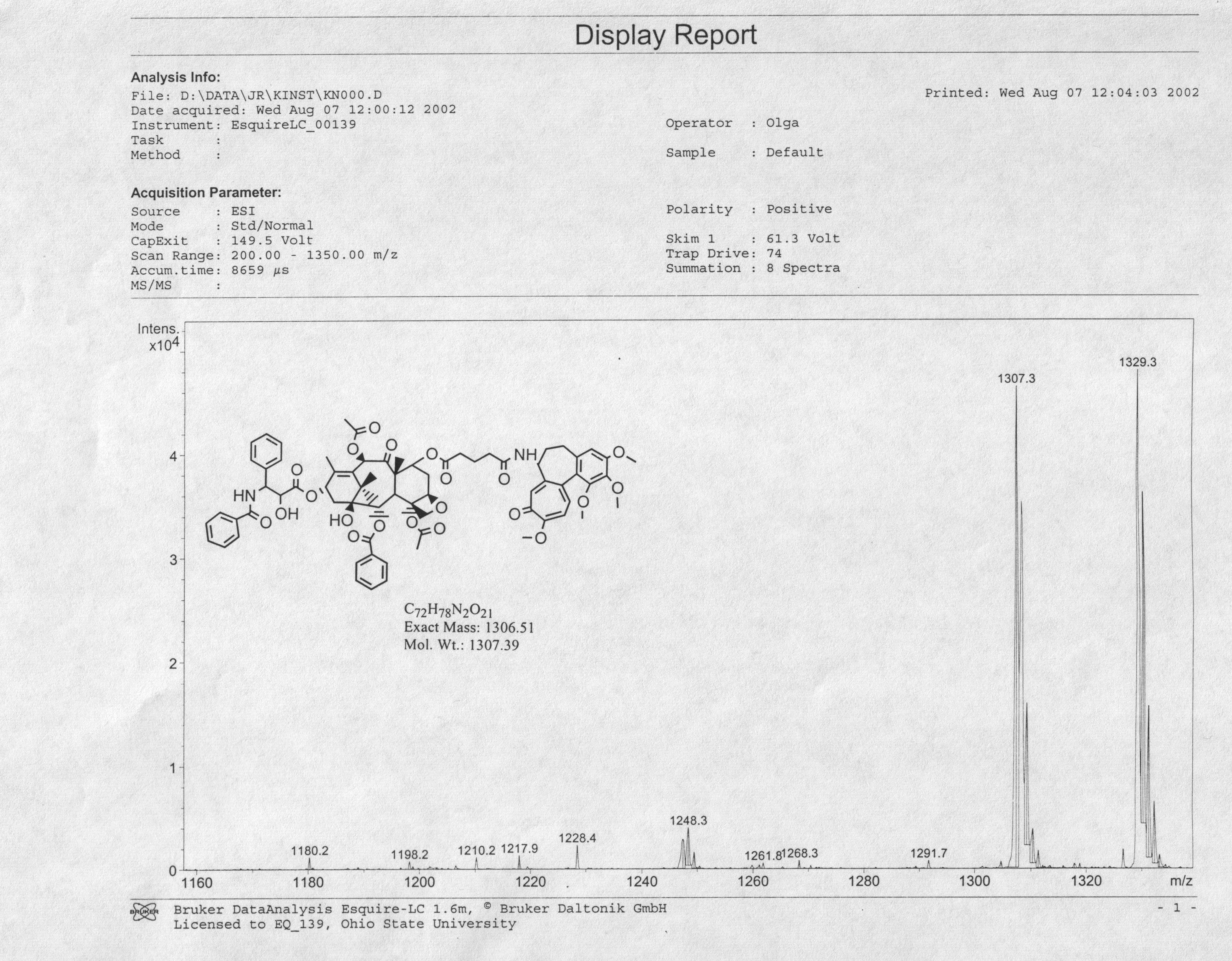

Supplement: File 2 — Mass Spectrum: Spectrum from Electron Spray Ionization Mass Spectrometry. [file Beilstein_J_Org_Chem-02-13-s002.jpeg]

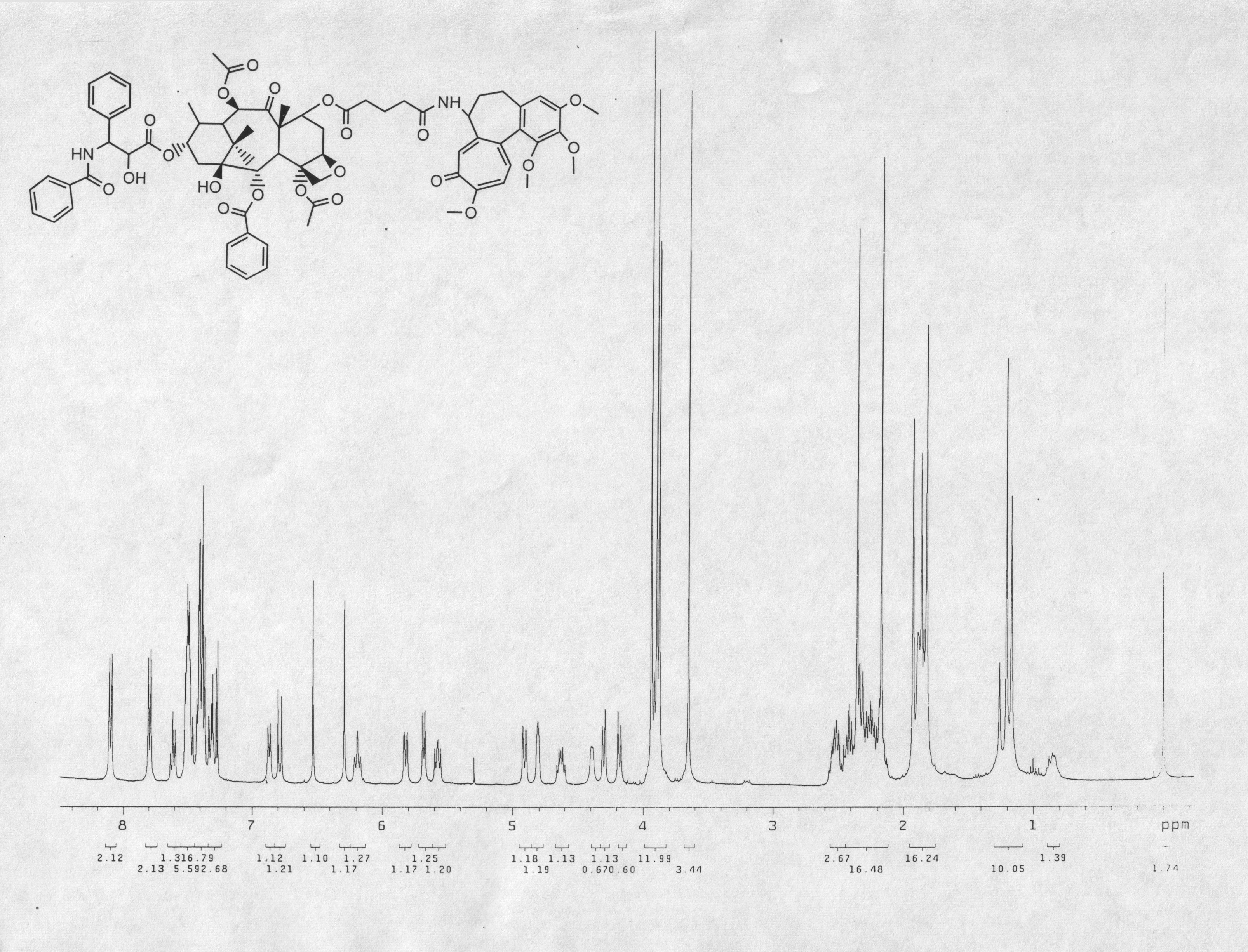

Supplement: File 3 — Proton NMR: Spectrum from Proton Nuclear Magnetic Resonance. [file Beilstein_J_Org_Chem-02-13-s003.jpeg]

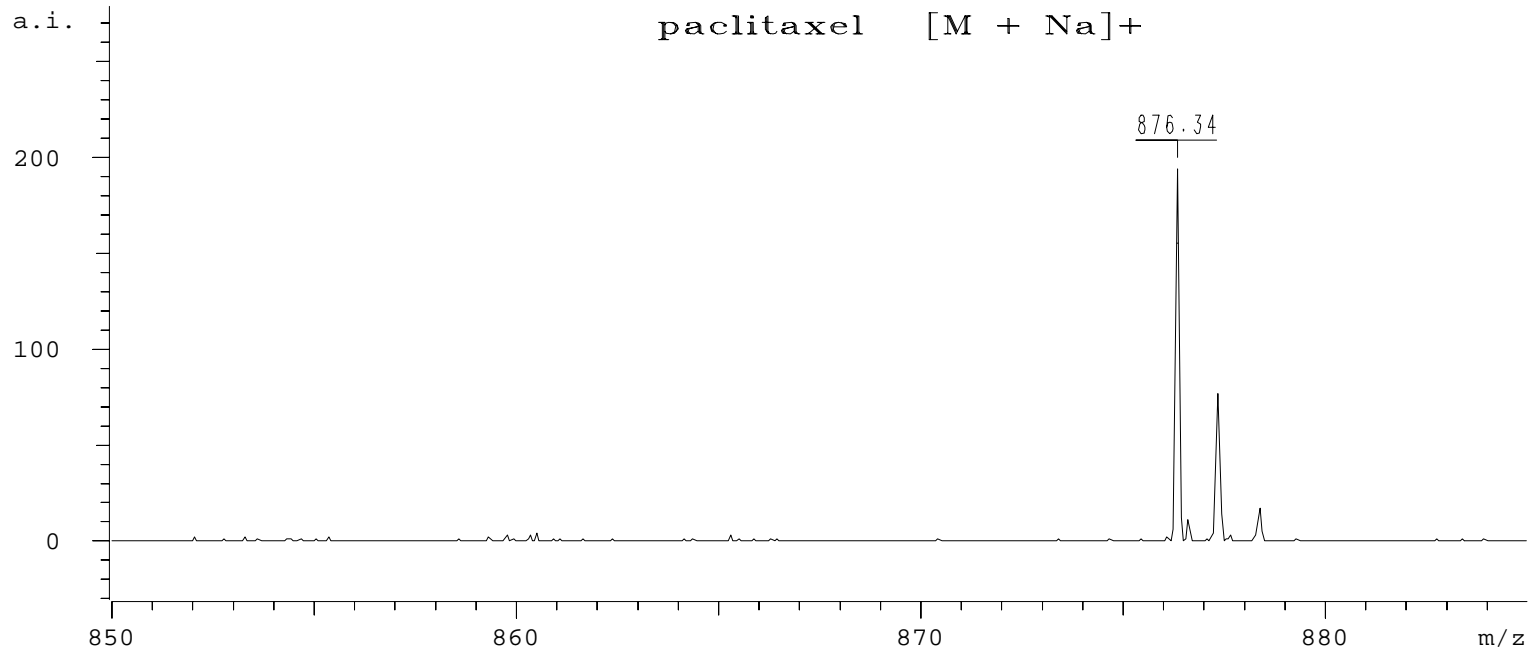

PULPROG  
TD 2048  
NS 16  
DS 0  
SW 20.000 ppm

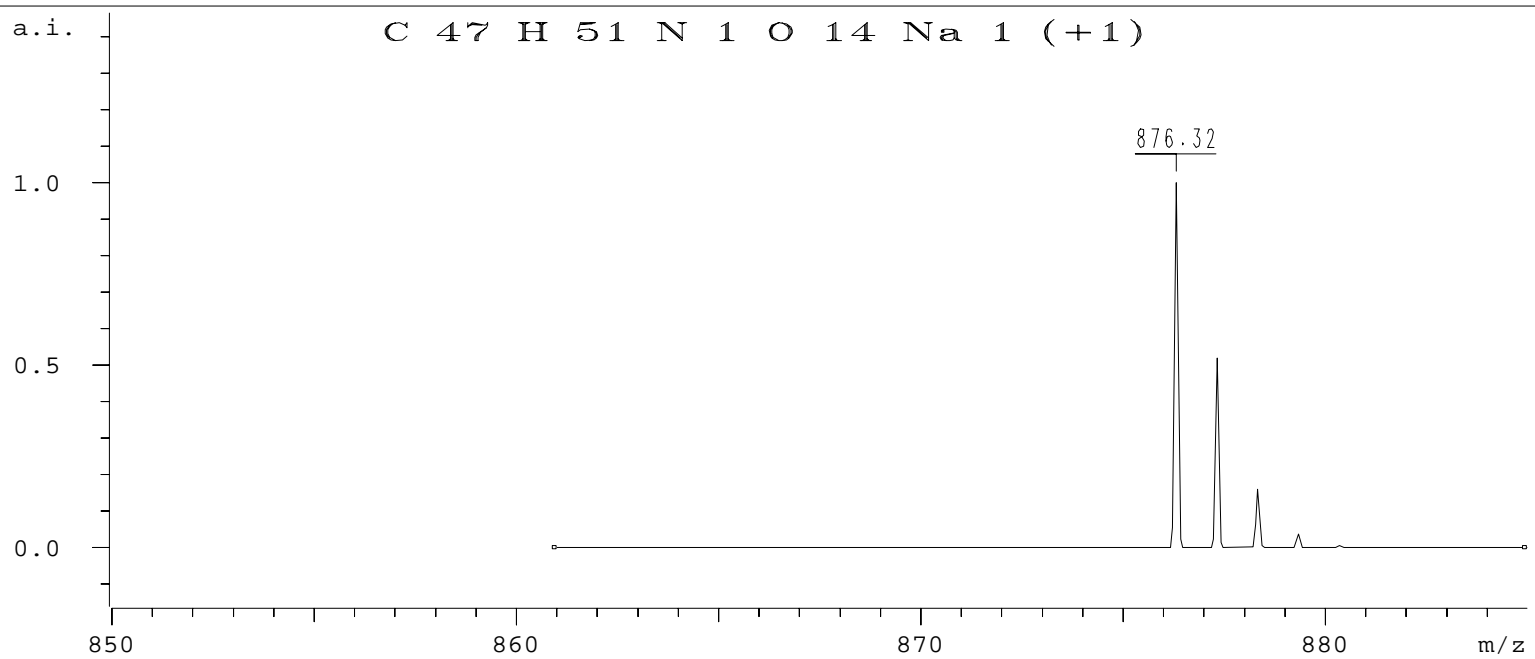

Supplement: File 7 — High-resolution MS of paclitaxel. (Top) Spectrum of starting material. (Bottom) Theoretical spectrum of paclitaxel sodium salt [file Beilstein_J_Org_Chem-02-13-s007.pdf]
