# Supplementary material for: Colchitaxel, a coupled compound made from microtubule inhibitors colchicine and paclitaxel
Source: Beilstein J Org Chem. 2006 Jun 30;2:13. doi: 10.1186/1860-5397-2-13 (PMC1557522; doi:10.1186/1860-5397-2-13)
Supplement: File 6 — High-resolution MS of colchicine. [file Beilstein_J_Org_Chem-02-13-s006.pdf]

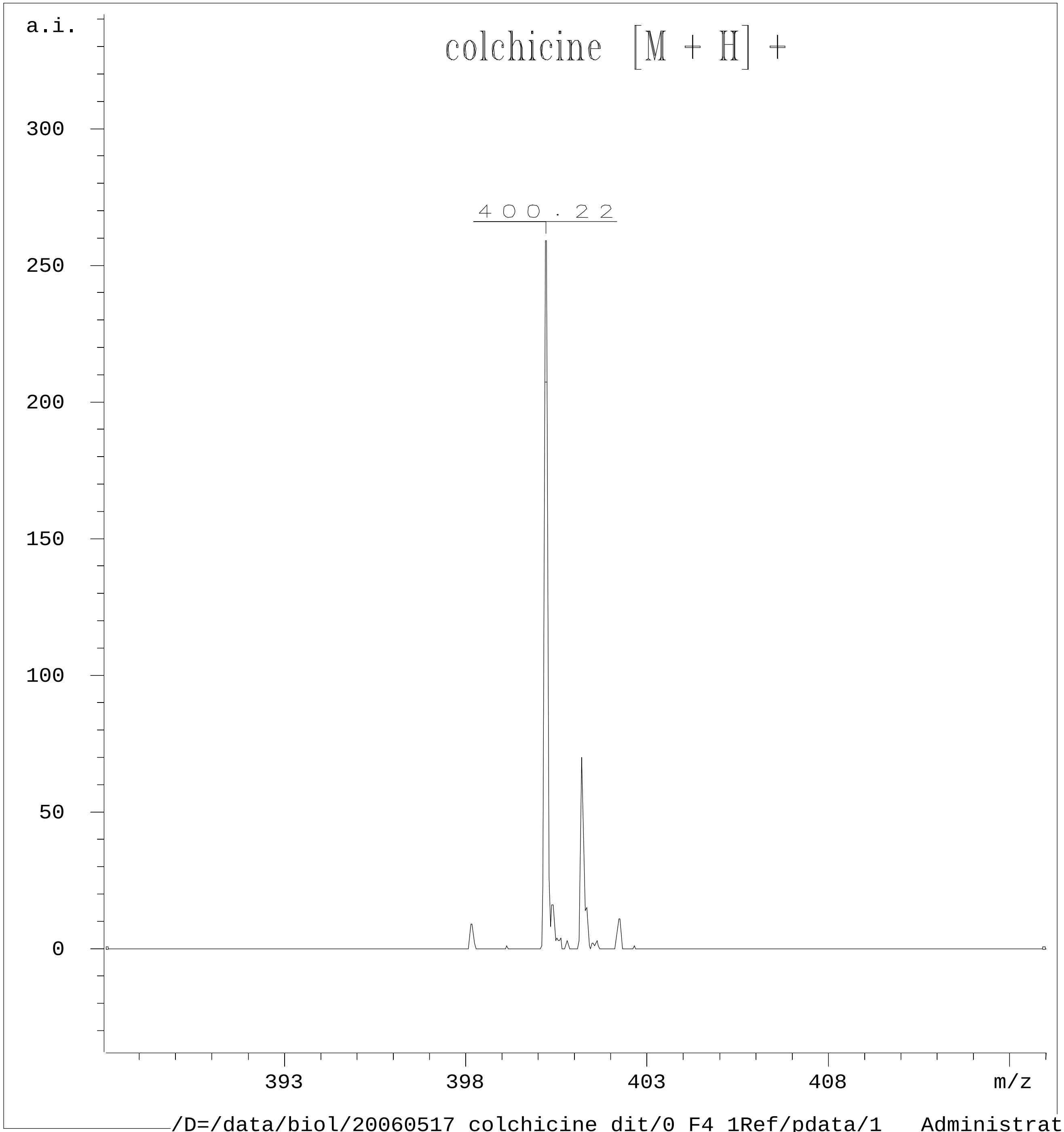


```
INSTRUM OMNIFLEX
OpId    tof
SMPNAM  20060517_colchi
AQ_DATE 2006-05-17 13:48:04
PATH    D:\data\biol
POLARI  POS
AQOP_m  Linear
TD           32683
NoSHOTS         60
SMONUM           0
SMOPTS1          0
SMOPTS2          0
SMOPTS3          0
DW               1.00  [ns]
DELAY      11513 [ns]
Uis1            19.00  [kV]
Uis2            13.95  [kV]
Urefl           20.00  [kV]
Ulens            9.20  [kV]
Uhimass          0.00  [kV]
RefFull          0.00  [kV]
UdetL            1.65  [kV]
UdetR            1.70  [kV]
Udefl            0.00  [kV]
REPHZ            5.00  [Hz]
ATTEN           33.0
ML1         786616.393
ML2            238.332
ML3              0.000
HITURBO no
GDEON   yes
GDEDLY  short
DEFLON  no
RLNSBND no
LLNSBND no
UIS2BND no
DPCAL1           0.38
DPMASS         500.00  [Da]
RBNDVAL          0.00
LBNDVAL          0.00
IS2BNDV          0.00
CMT1    FLEXControl generated XMASS data
CMT2    (c) 2000 Bruker Daltonics
```

/D=/data/biol/20060517_colchicine_dit/0_F4_1Ref/pdata/1   Administrator   Wed May 17 14:37:44 2006
