# Supplementary material for: Colchitaxel, a coupled compound made from microtubule inhibitors colchicine and paclitaxel
Source: Beilstein J Org Chem. 2006 Jun 30;2:13. doi: 10.1186/1860-5397-2-13 (PMC1557522; doi:10.1186/1860-5397-2-13)
Supplement: File 8 — Experimental data (DOC) containing all experimental data [file Beilstein_J_Org_Chem-02-13-s008.doc]

**Experimental**

**General Analytical Procedures**

Paclitaxel was obtained from Dabur, Inc., India. Colchicine was purchased from Acros Organics (Ceel, Belgium). Both were judged >97% pure after assessment by NMR, HPLC, and verification of m.p. All melting points were obtained using Mel-Temp apparatus. Proton and carbon NMR spectra were obtained in a 400 MHz Varian Unityplus NMR spectrometer. Tetramethylsilane (TMS) was used as an internal reference for NMR. IR spectra were recorded using a Thermo Nicolet IR 200 spectrophotometer. Mass spectra were recorded using a Shimadzu QP5050A/GC-17A instrument. All solvents were distilled prior to use. Flash column chromatography was carried out with Merck silica gel (60-200 Mesh size). Air-sensitive reactions were carried out under Ar or N_2_. The final product was stored under Ar at -70° C. Commercial programs, ClogP (BioByte, Claremont, CA) and LogD Suite from Advanced Chemistry Development (Toronto, Canada), were used to predict the partition coefficient of the final coupled compound **(1)**.

**Cell Culture**

The IAR20 PC1 line, derived from the liver of inbred BD-VI rats, was available from ATCC (Manassas, VA). These cells were routinely grown in William’s E medium supplemented with penicillin, streptomycin, 50 units/ml each, and 10% fetal bovine serum as described elsewhere [60]. Cells were subcultured weekly at a density sufficient to attain confluency within one week. The original standard curve relating cell shape features to cancer was developed by sampling and analyzing cells at various times over a time course of approximately 8 months [61, 62].

**Indirect Immunofluorescence Localization**

For immunofluorescence localization studies, cells were subcultured onto glass coverslips and left for 18-48 h to attach. They were treated with various compounds for 2 h. The coverslips were collected by immersion in methanol at -20 °C. N357 monoclonal antibody against β-tubulin (Amersham Biosciences, Piscataway, NJ) was diluted 1:600 for use in microtubule localizations. Mouse antibody against EB1, obtained from BD Transduction Laboratories (San Francisco, CA), was diluted 1:100. As secondary antibodies, FITC-conjugated (U. S. Biochemical Corp., Cleveland, OH) or Alexa 488-conjugated goat anti-mouse immunoglobulin G (Molecular Probes, Eugene, OR) were used as described previously [17]. Wide-field fluorescence images were recorded on a Zeiss Axiophot microscope equipped with a 63x Neofluor lens. Images were acquired from a Roper Scientific RTE/CCD camera (Tucson, AZ) and PC running MetaMorph 4.6r5 software (Universal Imaging Corp., Buckinghamshire, UK).

**Laser Confocal Scanning Microscopy (LCSM) and Visualization**

LCSM of the samples was performed on a Zeiss Pascal confocal microscope equipped with a 63x Neofluor lens. Software used for deconvolution included the Volocity (Improvision, Coventry, UK) and Huygens (Advanced Volume Imaging, Hilversum, The Netherlands) packages. VROs were made in VRWorx 2.5 software (VRToolbox, Pittsburgh, PA). The resulting three-dimensional images are displayed in Quickview on Apple Macintosh or Dell PC microcomputers.

**N-[(tert-butoxy)carbonyl]colchicine (3).** Di(*t*-butyl)dicarbonate (600 mg, 2.75 mmol) was added to a mixture of colchicine (200 mg, 0.5 mmol), Et_3_N (0.1 ml), and DMAP (68 mg) in 2 ml of CH_2_Cl_2_. The reaction mixture was stirred for 1 day and solvent removed in vacuum. Chromatographic separation of the residue produced 56 mg (23%) of N-[(tert-butoxy)carbonyl]colchicine as a yellow solid. m.p. 75-78 ^o^C. ^1^H-NMR (400 MHz, CDCl_3_): δ 1.4 (s, 9H, t-butyl), 1.85 (m, 2H, H-6), 2.5-2.8 (m, 2H, H-5, ), 2.25 (s, 3H, CH_3_), 3.7 (s, 3H, OCH_3_), 3.8 (s, 3H, OCH_3_), 3.8 (s, 3H, OCH_3_), 4.0 (s, 3H, OCH_3_), 5.14 (dd, 1H, J=5.7, 11.8, H-7), 6.53 (s, 1H, H-4), 6.78 (d, 1H, J=10.6, H-12), 7.21 (d, 1H, J=10.5, H-12), 7.58 (s, 1H, H-13) (Lit. [38])

**N-[(tert-butoxy)carbonyl]deacetylcolchicine (4).** 0.08 ml of methanol solution of MeONa (10% w/v) was added to a solution of **(3)** (48 mg, 0.1 mmol) in 1 ml of methanol at 0^o^C and stirred for 1 h. The mixture was neutralized with aqueous ammonium chloride, filtered, and solvents removed in vacuum. The residue was purified by chromatography to give 37.5 mg (82%) of N-[(tert-butoxy)carbonyl]deacetylcolchicine. m.p. 82-84 ^o^C (Lit. 80-84 ^o^C [42]). ^1^H-NMR (400 MHz, CDCl_3_): δ 1.4 (s, 9H, t-butyl), 1.7 (m, 2H, H-6), 2.4 (s, 2H, H-5), 3.7 (s, 3H, OCH_3_), 3.8 (s, 3H, OCH_3_), 3.8 (s, 3H, OCH_3_), 4.0 (s, 3H, OCH_3_), 4.4 (m, 1H, H-7), 5.1 (d, 1H, J=7.5, NH), 6.5 (s, 1H, H-4), 6.8 (d, 1H, J=10.6, H-12), 7.2 (d, 1H, J=10.6, H-12), 7.5 (s,1H, H-13) (Lit. [38])

**N-Deacetylcolchicine** **(5).** 0.1 ml of TFA was added to a solution of **(4)** (35 mg, 0.077 mmol) in 2 ml of CH_2_Cl_2_, and the reaction mixture was stirred for 3 h at room temperature. Solvent and excess TFA were removed, and the residue was redissolved in toluene, washed with aqueous ammonia, and solvent was removed again. The residue was purified by chromatography to produce 20 mg (73%) of N-deacetyl-colchicine **(5)**. m.p. 144-147 ^o^C (Lit. 141-146 °C, [42])

**N-Glutaryldeacetylcolchicine (6).** Glutaric anhydride (20 mg, 0.17 mmol) was added to a mixture of 60 mg (0.17 mmol) compound **(5)** and 1.75 ml DMF (0.22 mmol). The mixture was stirred at room temperature, diluted with 15 ml of ether, washed with water, and solvents were removed in vacuum. The crude product was purified by chromatography to give 45 mg (63%) of **6.** ^1^H-NMR (400 MHz, CDCl_3_): δ 1.9 (m, 2H, H-6), 2.1 (m, 2H, H-3'), 2.2 (t, 2H, J=9.0, H-2'), 2.4 (t, 2H, J= 8.0, H-5), 2.6 (t, 2H, J=9.0, H-4'), 3.6 (s, 3H, OCH3-10), 3.8 (s, 3H, OCH_3_-3), 3.9 (s, 3H, OCH_3_-2), 3.9 (s, 3H, OCH_3_-1), 4.6 (m, 1H, H-7), 6.5 (s, 1H, H-4), 6.9 (d, 1H, J=10.8, H-11), 7.4 (d, 1H, J=10.8, H-12), 7.7 (s, 1H, H-8), 8.7 (bs, OH). APT NMR (400 MHz, CDCl_3_) δ: 20.8 (CH_2_), 29.6 (CH_2_), 29.9 (CH_2_), 34.2 (CH_2_), 35.8 (CH_2_), 52.7 (CH), 56.0 (CH_3_), 56.4 (CH_3_), 61.3 (CH_3_), 61.6 (CH_3_), 107.3 (CH), 113.9 (CH), 125.3 (C), 130.8 (CH), 134.4 (C), 134.4 (C), 136.2 (CH), 137 (C), 141.4(C), 150.9 (C), 153.5 (C),153 .5 (C) 163.8 (C), 172.8 (C), 179.2 (C). (MS, m/z (rel. int.): 471 (30), 454 (40), 356 (35), 314 (100).

**2'-O*-*(*tert*-Butyldimethylsilyl)paclitaxel (8).** 352 mg (2.3 mmol) of TBDMSCl was added to a solution of paclitaxel (200 mg, 0.23 mmol) and ten-fold molar excess of DMAP in 2 ml of dry CH_2_Cl_2_. The reaction mixture was stirred for 12 h at room temperature, quenched with 30 ml of saturated aqueous NH_4_Cl, and extracted into CH_2_Cl_2_. Organic layer was dried over MgSO_4_, the solvent removed in vacuum, and the solid residue was separated by column chromatography to give 150 mg (67%) of 2'-O*-*(*tert*-butyldimethylsilyl)paclitaxel **(8)**. m.p. 162-163 ^o^C. ^1^H-NMR (400 MHz, CDCl_3_): δ -0.28 (s, 3H, CH_3_-Si), -0.04 (s, 3H, CH_3_-Si), 0.79 (s, 9H, t-butyl), 1.12 (s, 3H, CH_3_-17), 1.22 (s, 3H, CH_3_-17), 1.67 (s, 3H, CH_3_-19), 1.89 (s, 3H, CH_3_-18), 2.15 (dd, 1H, J=8, H-6α), 2.20 (s, 3H, 4-OCOCH_3_), 2.15 (dd, 1H, J=8, H-6β), 2.54 (m, 2H, H-14), 2.56 (s, 3H, 10-OCOCH_3_), 3.80 (d, 1H, J=6.8, H-3), 4.21 (d, 1H, J=8.4, H-20α), 4.29 (d, 1H, J=8.4, H-20β), 4.42 (m, 1H, H-7), 4.96 (d, 1H, J=8.41, H-5), 5.67 (d, 1H, J=7.2, H-2^*^), 5.74 (dd, 1H, J=8.8, 2.4, H-3^*^), 6.28 (t, 1H, J=8.8, H-13), 6.30 (s, 1H, H-10), 7.15-7.65 (m, 11H, Ar-H), 7.75 (dd, 2H, J=7.2, 1.2, *o-*H in 2-benzo group), 8.15 (dd, 2H, J=7.2, 1.2, *o-*H in 4'- benzo group) (Lit. [43])

**2**’**-O-(*tert-*Butyldimethylsilyl)colchitaxel (9).**  DCC (52.4 mg, 0.25 mmol) was added to a solution of 0.06 mmol DMAP, 2'-O*-*(*tert*-butyldimethylsilyl)paclitaxel (**8**, 116 mg, 0.12 mmol) and *N*-glutaryldeacetylcolchicine (**6**, 56.5 mg, 0.12 mmol) in 3 ml of dry CH_2_Cl_2_. The resultant mixture was stirred for 24 h, and solvent removed in vacuum. Crude **9** was purified by chromatography to produce 80 mg (48%) of the pure product 2’-O-(*tert-*butyldimethylsilyl) colchitaxel **(9)**. m.p. 183-185 ^0^C. ^1^H-NMR (400 MHz, CDCl_3_): δ -0.28 (s, 3H, CH_3_-Si), -0.04 (s, 3H, CH_3_-Si), 0.80 (s, 9H, t-butyl), 1.16 (s, 3H, CH_3_-17), 1.22 (s, 3H, CH_3_-17), 1.69 (s, 3H, CH_3_-19), 1.90 (s, 3H, CH_3_-18), 2.10 (dd, 1H, J=8, H-6α), 2.22 (s, 3H, C-4-OCOCH_3_), 2.34 (dd, 1H, J=8.0, H-6β), 2.54 (m, 2H, H-14), 2.57 (s, 3H, C-10-OC(O)CH_3_), 3.66 (s, 3H, OCH_3_), 3.90 (s, 3H, OCH_3_) 3.94 (s, 3H, OCH_3_), 3.98 (s, 3H, OCH_3_), 4.18 (d, 1H, J=8.4, H-20α), 4.30 (d, 1H, J=8.4, H-20β), 4.4 (m, 1H, H-7), 4.82 (m, 1H, H-2^*^), 4.62(m, 1H, H-7'), 4.92 (d, 1H, J=8.4, H-5), 5.60 (m, 1H, NH), 5.72 (d, 1H, J=7.2, H-2), 5.78 (dd, 1H, J=8.8, 2.4, H-3^*^), 6.21 (t, 1H, J=8.8, H-13), 6.38 (s, 1H, H-10), 6.52 (s, 1H, H-4'), 6.78 (d, 1H, J=10.8, H-12'), 6.86 (d, 1H, J=10.8, H-11'), 7.25(d, J=8.2, NH), 7.30-7.65 (m, 15H, Ar-H), 7.75 (dd, 2H, J=7.2, 1.2, *o*-H in 2-benzo group), 8.15 (dd, 2H, J=7.2, 1.2, *o*-H in 4'- benzo group).

**Colchitaxel (1)**. A solution of 2’-O-(*tert-*butyldimethylsilyl) colchitaxel (**9**, 50 mg, 0.036 mmol) in 5 ml of tetrahydrofuran was treated with 18 mg TBAF in at 0^o^C for 5 min and at 25^o^C for 45 min. Solvent was removed in vacuum and crude **1** was purified by chromatography to give 25 mg (55%) of pure colchitaxel **(1)**. m.p. 165-168 ^o^C.

**Chemical Analysis**

Analysis of the purity of colchitaxel by HPLC indicated that it was the major component and constituted 98% of the sample. The mass of colchitaxel was confirmed by electron spray ionization-mass spectrometry as 1307.3, consistent with structure **(1).** Further structural analysis was done by proton NMR and the attached proton test. See additional files 1-3 for the original spectra. The results were consistent with the structure shown. Both electron spray ionization and chemical ionization produced values of molecular weight +=1307, consistent with structure **(1)**. HRMS (M+1): exper. 1307.5175, calc. C_72_H_79_N_2_O_21_ 1307.5175. ^1^H-NMR (400 MHz, CDCl_3_): δ 1.16 (s, 3H, CH_3_-17), 1.22 (s, 3H, CH_3_-17), 1.82 (s, 3H, CH_3_-19), 1.94 (s, 3H, CH_3_-18), 2.20 (s, 3H, 4-OCOCH_3_), 2.25 (dd, 1H, J=8, H-6α), 2.36 (s, 3H, 10-OCOCH_3_), 2.40 (dd, 1H, J=8.0, H-6β), 2.54 (m, 2H, H-14), 3.66 (s, 3H, OCH_3_), 3.88 (s, 3H, OCH_3_), 3.90 (s, 3H, OCH_3_), 3.94 (s, 3H, OCH_3_), 4.18 (d, 1H, J=8.4, H-20α), 4.30 (d, 1H, J=8.4, H-20β), 4.4 (m, 1H, H-7), 4.62 (m, 1H, H-7'), 4.82 (m, 1H, H-2^*^), 4.92 (d, 1H, J=8.4, H-5), 5.58 (m, 1H, NH), 5.68 (d, 1H, J=7.2, H-2), 5.82 (dd, 1H, J=8.8, 2.4, H-3^*^), 6.18 (t, 1H, J=8.8, H-13), 6.30 (s, 1H, H-10), 6.52 (s, 1H, H-4'), 6.78 (d, 1H, J=10.8, H-12'), 6.86 (d, 1H, J=10.8, H-11'), 7.25(d,1H, J=7.8, NH), 7.30-7.65 (m, 15H, Ar-H), 7.75 (dd, 2H, J=7.2, 1.2, *o*-H in 2-benzo group), 8.15 (dd, 2H, J=7.2, 1.2, *o*-H in 4'- benzo group).

(APT) NMR (400 MHz, CDCl_3_) δ: 11.28 (CH_3_, C-19), 15.00 (CH_3_, C-18), 20.9 (CH_2_, C-3''), 21.15 (CH_3_, C-17), 21.33 (CH_3_, C-4-OC(O)CH_3_), 22.85 (CH_3_, C-10-OC(O)CH_3_), 26.79 (CH_3_, C-16), 30.31 (CH_2_, C -5'), 33.65 (CH_2_, C -2''), 33.77 (CH_2_, C -4''), 35.59(CH_2_, C -14), 36.02 (CH_2_, C -6'), 31.17 (CH_2_, C -6), 43.55 (C, C -15), 47.31 (CH, C -3), 52.35 (CH, C -3*), 55.45 (OCH_3_, C-1'), 56.48 (2xOCH_3_, C-2', 3' ), 56.66 (C, C -8), 61.70 (CH, C-7'), 61.88 (OCH_3_, C-10'), 71.96 (CH, C -7), 72.14 (CH, C -13), 73.78 (CH, C -2^*^), 74.75 (CH, C -2), 75.66 (CH, C -10), 76.75 (CH_2_, C-20), 78.94 (C, C -1), 81.24 (C, C-4), 84.16 (CH, C -5), 107.77 (CH, C -4'), 112.57 (CH, C -11'), 126.10-141.16 (26xC, Ar-CH), 151.72 (3xC, C -1', C -2', C -3'), 153.84 (C, C -10'), 164.34 (C=O, C -5''), 167.20 (C=O, C -4*), 167.50 ((C-2)- O-C=O), 169.56 (C, C -1''), 170.66 ((C-4)-O-C=O), 172.42 ((C-10)- O-C=O),172.53 (C=O, C-1^*^) 179.88 (C, C-9'), 202.71 (C, C -9).

60. Montesano R, Saint Vincent L, Drevon C, Tomatis L: *Int. J. Cancer* 1975, **16:**550-558.

61. Heckman CA: **Cell shape and growth control.** In *Advances in Cell Culture.* Edited by Maramorosch K. New York: Academic Press, 1985; 85-156 [Vol. 4].

62. Heckman CA, Campbell AE, Wetzel B: Exp. Cell Res. 1987, **169:**127-148.
